# Single-step RT-PCR assay for dual genotyping of GI and GII norovirus strains

**DOI:** 10.1016/j.jcv.2020.104689

**Published:** 2021-01

**Authors:** Preeti Chhabra, Hannah Browne, Thalia Huynh, Marta Diez-Valcarce, Leslie Barclay, Margaret N. Kosek, Tahmeed Ahmed, Maria Renee Lopez, Chao-Yang Pan, Jan Vinjé

**Affiliations:** a Viral Gastroenteritis Branch, Division of Viral Diseases, Centers for Disease Control and Prevention, Atlanta, GA, USA; b National Foundation for the Centers for Disease Control and Prevention Inc., Atlanta, GA, USA; c California Department of Public Health, Richmond, CA, USA; d Rollins School of Public Health, Emory University, Atlanta, GA, USA; e University of Virginia Division of Infectious Diseases and International Health, Charlottesville, VA, USA; f International Centre for Diarrhoeal Disease Research, Dhaka, Bangladesh; g Universidad del Valle de Guatemala, Guatemala City, Guatemala

**Keywords:** Norovirus, Gastroenteritis, RT-PCR

## Abstract

**Background:**

Noroviruses are the major cause of acute gastroenteritis (AGE) in people of all ages globally. Standardized genotyping is key for outbreak investigations and surveillance networks.

**Objective:**

Here we describe the validation of a one-step conventional RT-PCR assay for sequence-based dual typing of GI and GII noroviruses.

This polymerase (P) and capsid (C) dual typing assay uses a combination of previously published oligonucleotide primers amplifying a genomic region spanning the 3'-end of ORF1 and 5'end of ORF2 resulting in a 579 bp product for GI and 570 bp product for GII viruses.

**Results:**

The limit of detection of the assay ranged from 5 to 50 copies of viral RNA per reaction for GI and GII. To validate the assay, we tested 2,663 noroviruspositive stool samples from outbreaks and sporadic cases of AGE in Bangladesh, Guatemala, Peru, and USA collected between 2010–2019, of which 2,392 (90 %) were genotyped successfully. Most of the known genotypes infecting humans (GI (n = 9) and GII (n = 23)) and P types (GI (n = 15), GII, (n = 20)) could be detected. The remaining 270 samples had low viral load (Ct > 30) by real-time RT-PCR. A panel of 166 samples positive for other enteric viruses (rotavirus, astrovirus, sapovirus, adenovirus type 40/41) tested negative.

**Conclusion:**

The use of broadly reactive genotyping assays greatly strengthens exchange of standardized genotype data globally to monitor trends in genotype diversity which is important for both the development of vaccines and to measure their impact.

## Highlights

• We validated genogroup-specific one-step conventional RT-PCR assays (PC assays) for sequence-based dual typing of GI and GII norovirus strains.
• The PC assays use a combination of oligonucleotide primers that target a genomic region spanning the 3’-end of ORF1 and 5’end of ORF2 of GI and GII noroviruses.
• The PC assays are sensitive (5 to 50 copies/rx) and detect all currently identified norovirus P-types and capsid genotypes from different geographic regions.
• The PC assays have been successfully implemented by CaliciNet USA and CaliciNet China.

## 1 Introduction

Human noroviruses are a leading cause of acute gastroenteritis in all age groups, globally [1]. Vaccines are under development which, if effective, offer promise to prevent hundreds of millions of gastroenteritis cases annually [2]. The viral genome is divided into three open reading frames (ORFs) with ORF1 encoding the nonstructural viral proteins including the RNA-dependent RNA polymerase (RdRp), ORF2 and ORF3 encoding the major (VP1) and minor (VP2) capsid proteins, respectively.

Genetically, norovirus can be divided into 10 genogroups (G) of which viruses from GI and GII cause almost all infections in humans. They can be further segregated into 35 genotypes based on amino acid diversity of VP1 [3]. In addition, the RdRp region of GI and GII noroviruses can be divided into 51 P-types based on nucleotide diversity [3]. Since the recognition that noroviruses frequently recombine at ORF1-ORF2 junction [4,5], dual typing of norovirus strains improves the ability to correctly identify strains [6].

Many laboratories perform norovirus typing by sequencing partial polymerase and capsid regions using two separate RT-PCR methods [[7], [8], [9], [10]]. To simplify the protocol, we combined existing oligonucleotide primers targeting the 3’-end of ORF1 and 5’-end of ORF2 [11] and validated the GI and GII specific conventional RT-PCR assays using a large panel of norovirus-positive stool samples.

## 2 Materials and methods

**Fig. 1:**
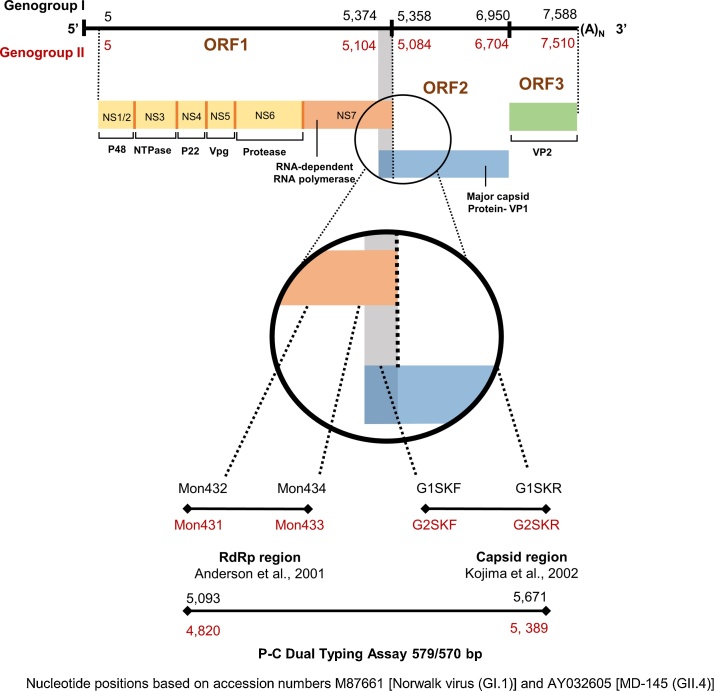
Genomic region amplified by one-step RT-PCR dual typing assays. The location of open reading frames corresponds to the genome organization of Norwalk virus (M87661) for GI and MD145 virus (AY032605) for GII viruses. This figure was created with BioRender.com.

The genogroup-specific polymerase – capsid typing (PC) assays were developed using a combination of previously published oligonucleotide primers [12,13] including MON432 (TGG ACI CGY GGI CCY AAY CA) targeting a small region at the 3’-end of ORF1 (polymerase), and G1SKR (CCA ACC CAR CCA TTR TAC A), targeting a region of the 5’-end of ORF2 for GI viruses and oligonucleotide primers MON431 (TGG ACI AGR GGI CCY AAY CA) and G2SKR (CCR CCN GCA TRH CCR TTR TAC AT) for GII viruses resulting in a 579 bp product for GI and 570 for GII viruses (Fig. 1) [14]. The limit of detection (LOD) of the assays were determined by analyzing 5 μl of 10-fold serial dilutions of quantified RNA transcripts of norovirus GI.1 and GII.4 Sydney ranging from 10^−1^ to 10^8^ copies/μl. We used a panel of 166 stool samples positive for other viruses including rotavirus (n = 48), sapovirus (n = 41), astrovirus (n = 39) and adenovirus 40/41 (n = 38) to determine the specificity of the assays [14]. To validate PC assays, we tested 2,663 norovirus-positive stool samples from different outbreaks and sporadic cases of acute gastroenteritis from Bangladesh (n = 91), Guatemala (n = 61), Peru (n = 1,288), and USA (n = 1,223) collected between 2010 and 2019 [[15], [16], [17]]. CDC Human Research Protection Office determined the study as public health non-research therefore human subject regulations did not apply. Viral nucleic acid was extracted from 10 % clarified fecal suspensions prepared in phosphate buffered saline using MagMax-96 Viral RNA Isolation Kit (Ambion, Foster City, CA, USA) according to the manufacturer's instructions on an automated KingFisher extractor (Thermo Fisher Scientific, Pittsburgh, PA, USA). After each sample was mixed with lysis buffer, coliphage MS2 virus was added as an RNA extraction control [14]. Viral RNA was detected using a multiplex real-time RT-PCR assay [11,18] with the AgPath-ID One Step RT PCR kit (Thermo Fisher Scientific, Pittsburgh, PA, USA).

PC assays was performed using Qiagen One-Step RT-PCR (Qiagen, Germantown, MD, USA) kit with 20 U of RNase inhibitor (Applied Biosystems, Foster City, CA, USA). Cycling conditions included reverse transcription at 42 °C for 30 min, activation of Taq polymerase at 95 °C for 15 min, and 40 cycles of PCR amplification at 95 °C, 50 °C, and 72 °C for 1 min each, followed by 10 min at 72 °C and cooling down to 4 °C. PCR products were visualized on a 1x TAE 2% agarose gel (Seakem-ME, Lonza, Allendale, NJ, USA) containing 10 μl of Gel Red (Biotium, Fremont, CA, USA), purified by ExoSAP-IT (Affymetrix, USB, Cleveland, OH, USA) or by a QIAquick PCR purification kit (Qiagen) followed by sequencing of the purified PCR products Eurofins MWG Operon, Louisville, KY, USA). Genotypes were assigned using an online human calicivirus typing tool (https://norovirus.ng.philab.cdc.gov/) [3].

## 3 Results

**Fig. 2:**
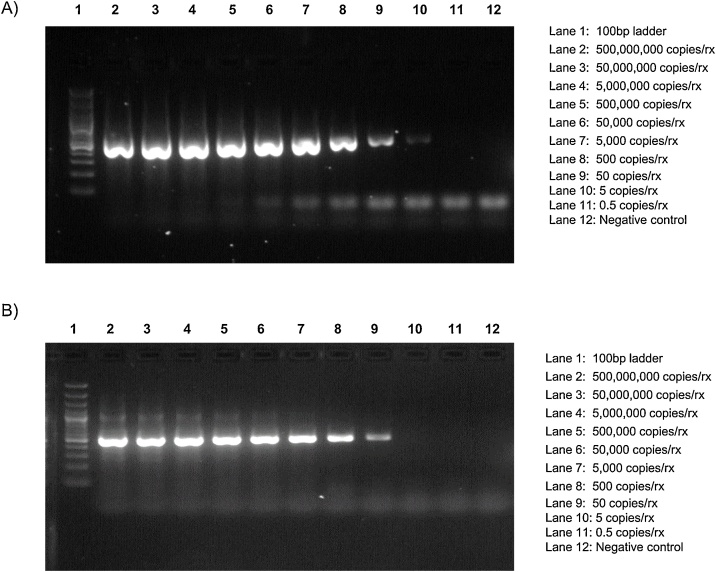
Ten-fold serial dilution of: (A) GI.1 RNA transcripts and (B) GII.4 Sydney RNA transcripts. PCR products were visualized on a 1 × TAE 2 % agarose gel containing Gel Red.

**Fig. 3:**
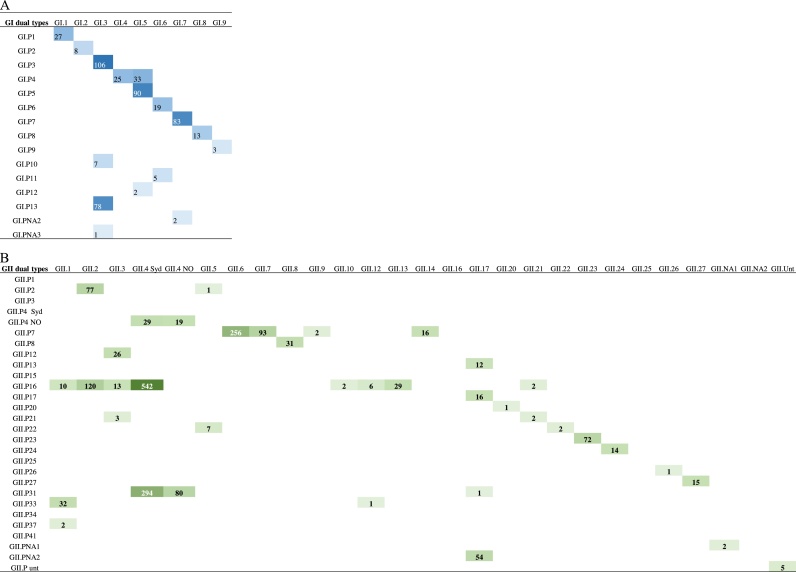
Norovirus polymerase and capsid dual type combinations of (A) genogroup I (n = 502) and (B) genogroup II (n = 1,890). The number of sequences available for each combination were grouped into quartiles separately for GI and GII. Quartiles with each dual type combination were marked blue for GI and green for GII. The raw data for this figure are listed in Table S1.

The LOD of the PC assays were 5 RNA copies/rx for GI and 50 copies/rx for GII (Fig. 2). All 166 stool samples positive for other gastroenteritis viruses tested negative (data not shown). A total of 2,392 (90 %) of the 2,663 norovirus-positive stool samples were successfully genotyped. The typed samples included all but one of the known GI and GII genotypes infecting humans (GI (n = 9) and GII (n = 23)) and P types (GI (n = 15), GII (n = 20)) (Fig. 3) (Table S1, S2 and S3). The 270 samples that could not be typed had a low viral load (Ct > 30) by real-time RT-PCR.

## 4 Conclusions

To harmonize genotyping of noroviruses globally, it is important that laboratories use robust and easily implementable genotyping protocols that amplify the same regions of the genome. Recently, we reported genogroup-specific one-step conventional RT-PCR assays for sequence-based dual typing of GI and GII noroviruses that amplify a small region at the 3’-end of ORF1 and a small region at the 5’-end of ORF2 [11]. In the current study, we evaluated the performance of these assays with a norovirus-positive stool panel including samples from Bangladesh, Guatemala, Peru, and the USA and demonstrated that all norovirus genotypes and P-types included in the panel could be detected.

Genotyping of norovirus strains for routine surveillance is based on conventional RT-PCR followed by Sanger sequencing. Since the mid-1990s, when detection and typing of noroviruses was primarily targeting a small region in the center of the polymerase gene [19,20], genotyping has evolved from a region at the 3’-end of ORF1 [12] to a region at the 5’-end of ORF2 [13], which in the last decade has become the typing assay used by many laboratories globally [7,[21], [22], [23], [24], [25]].

Protocols for typing both the polymerase and the capsid gene include the use of two different RT-PCR assays or amplification of a longer region of the genome which often includes a nested amplification step to increase sensitivity [[7], [8], [9], [10],^26^].

We recently successfully implemented these dual typing assays in several CaliciNet laboratories and after pilot data showed robust performance, the assays were officially adopted by the entire CaliciNet USA network [11] and by CaliciNet China [24]. A similar approach for genotyping of GI and GII norovirus strains has been reported by the Australian and New Zealand norovirus surveillance network [22,27].

The genogroup-specific PC assays have several limitations. First, the reverse GI and GII oligonucleotide primer sequences are quite similar and therefore the assays are not 100 % genogroup-specific. Consequently, samples should be first tested by GI/GII real-time RT-PCR to identify the genogroup of a strain prior to select if a GI or a GII PC assay should be employed for typing. Finally, samples with a low viral load (Ct values >30) are not robustly amplified and therefore the success rate of typing these strains is often low.

In summary, the norovirus PC assays are sensitive and detect all currently identified norovirus P-types and capsid genotypes from different geographic regions. The use of broadly reactive genotyping assays greatly strengthens exchange of standardized genotype data globally to monitor trends in genotype diversity which is important for both the development of vaccines and to measure their impact.

## Funding

This study was partly supported by a grant from the intramural food safety program of CDC (PC, HB, LB, JV) and in part by the Bill & Melinda Gates Foundation (OPP1066146) and 47075 to the Fogarty International Center (MNK). The funders had no role in study design, data collection and analysis, decision to publish, or preparation of the manuscript.

## Ethical approval

CDC Human Research Protection Office determined the study as public health non-research therefore human subject regulations did not apply.

## Disclaimer

The findings and conclusions in this article are those of the authors and do not necessarily represent the official position of the Centers for Disease Control and Prevention.

## CRediT authorship contribution statement

**Preeti Chhabra:** Conceptualization, Investigation, Writing - original draft. **Hannah Browne:** Investigation, Data curation. **Thalia Huynh:** Investigation, Data curation. **Marta Diez-Valcarce:** Visualization, Investigation, Writing - review & editing. **Leslie Barclay:** Data curation, Writing - review & editing. **Margaret N. Kosek:** Writing - review & editing. **Tahmeed Ahmed:** Writing - review & editing. **Maria Renee Lopez:** Investigation, Writing - review & editing. **Chao-Yang Pan:** Writing - review & editing. **Jan Vinjé:** Conceptualization, Supervision, Writing - review & editing.

## Declaration of Competing Interest

The authors report no declarations of interest.
